# Vaginal Anaplastic Lymphoma Kinase (ALK)-Positive Mesenchymal Tumor in a Young Patient: Expanding the Spectrum of Rare Genitourinary Neoplasms

**DOI:** 10.7759/cureus.94499

**Published:** 2025-10-13

**Authors:** D. Hassan, S. Percholli Ramasubramanian, B. Iqbal

**Affiliations:** 1 Obstetrics and Gynaecology, Ain Shams General Hospital, Cairo, EGY; 2 Obstetrics and Gynaecology, Wythenshawe Hospital, Manchester, GBR; 3 Obstetrics and Gynaecology, The Countess of Chester Hospital, Chester, GBR

**Keywords:** alk-positive, inflammatory myofibroblastic tumor, mesenchymal tumors, primary vaginal tumor, vaginal mass

## Abstract

Primary vaginal tumors are uncommon, and mesenchymal neoplasms in this location are even rarer, often resembling benign lesions such as fibroids or cysts. We present the case of a 20-year-old woman with a steadily enlarging anterior vaginal wall mass, initially suspected to be a fibroid based on clinical and radiological findings. Surgical excision revealed a spindle-cell proliferation with inflammatory infiltrates, and immunohistochemistry confirmed an anaplastic lymphoma kinase (ALK)-positive mesenchymal tumor. Postoperative imaging showed no residual disease, and a multidisciplinary team recommended surveillance given the complete excision and proximity of the lesion to the bladder trigone. This case emphasizes three clinical considerations: the importance of including rare mesenchymal tumors in the differential diagnosis of vaginal wall masses, the indispensable role of histopathology and immunohistochemistry in establishing a definitive diagnosis, and the need for tailored management strategies that balance oncologic control with preservation of function.

## Introduction

Recent evidence highlights the importance of differentiating benign-appearing vulvovaginal masses from rare neoplastic lesions, as such presentations may encompass a wide spectrum of pathologies beyond the commonly encountered Bartholin cysts and abscesses [1]. Primary vaginal cancer is rare, accounting for approximately 1-2% of all malignancies of the female genital tract. Many vaginal lesions are metastatic rather than primary. Among clinically encountered vaginal masses, benign entities such as Gartner duct cysts, leiomyomas, and endometriotic nodules are predominant. These benign lesions often present on the anterior vaginal wall and can mimic other gynecologic conditions [2,3].

Mesenchymal tumors that develop in the vagina are exceedingly rare, with primary vaginal sarcomas constituting a minor proportion of vaginal malignancies, which themselves are infrequent. The diagnosis of these lesions presents challenges due to their morphological and clinical similarities to more prevalent benign formations, such as fibroids, cysts, or endometriotic deposits, thereby necessitating meticulous pathological examination [4,5].

Anaplastic lymphoma kinase (ALK) rearrangements or overexpression are implicated in a significant proportion of inflammatory myofibroblastic tumors (IMTs), a mesenchymal neoplasm exhibiting intermediate biological behavior, with approximately 50-70% demonstrating ALK fusions identifiable through immunohistochemistry or molecular testing. Immunohistochemistry plays a crucial role in diagnosis, with ALK immunohistochemistry serving as the primary modality for confirming IMT and informing targeted therapeutic strategies when results are positive [6].

Our report of this case highlights the exceptional rarity of an ALK-positive mesenchymal tumor in the anterior vaginal wall of a young patient, a site rarely documented in existing literature. This case underscores the diagnostic challenges associated with such tumors, as they often resemble benign conditions like fibroids or cysts. This stresses the critical role of histopathology and immunohistochemistry in making a definitive diagnosis. Identifying spindle-cell morphology with inflammatory infiltrates and ALK positivity is crucial, with molecular confirmation sometimes required.

## Case presentation

We report the case of a 20-year-old woman, gravida 0 para 0+1 (having had a previous terminated pregnancy), who visited the gynecology outpatient clinic with a one-month history of a steadily growing vaginal lump. The lump was first noticed when she inserted a copper intrauterine device in March 2024. The mass was causing painful sex and a sudden need to urinate. Her past history was notable for dysmenorrhea and menorrhagia, managed with tranexamic acid and mefenamic acid, and a family history of breast cancer (aunt and grandmother). She had no prior cervical smear tests and was an active smoker.

**Figure 1:**
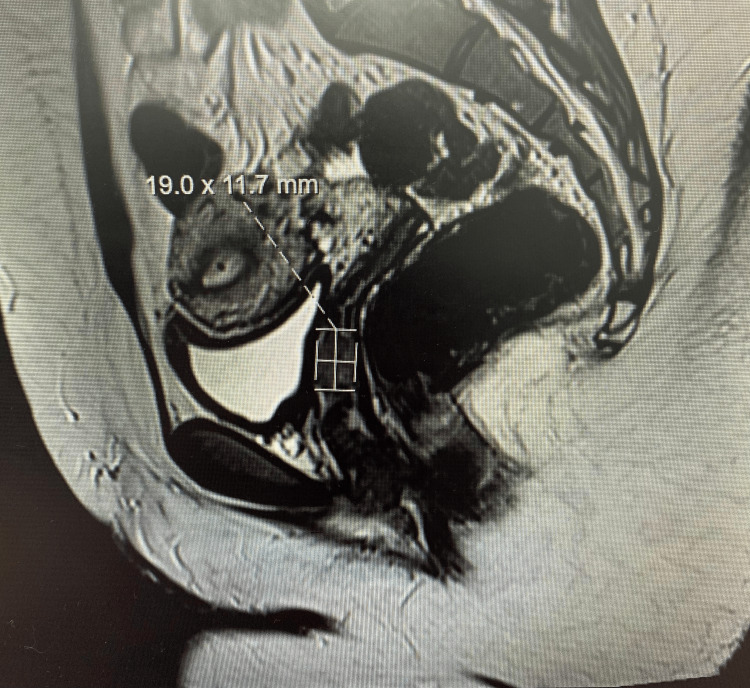
Pelvic MRI showing a well-defined mass initially resembling a uterine fibroid. Subsequent histopathology confirmed an ALK-positive mesenchymal tumor. MRI: magnetic resonance imaging; ALK: anaplastic lymphoma kinase

Clinical examination revealed a 2.5 cm firm mass arising from the anterior vaginal wall, with a normal cervix and anteverted uterus. Pelvic MRI demonstrated a 19 × 11.7 mm lesion arising from the mid-anterior vaginal wall with indeterminate T2 characteristics, not typical for endometriotic deposits, and no bladder invasion. Figure 1 shows preoperative MRI sagittal pelvic diagnostic imaging. Direct visualization and excision were advised.

**Figure 2:**
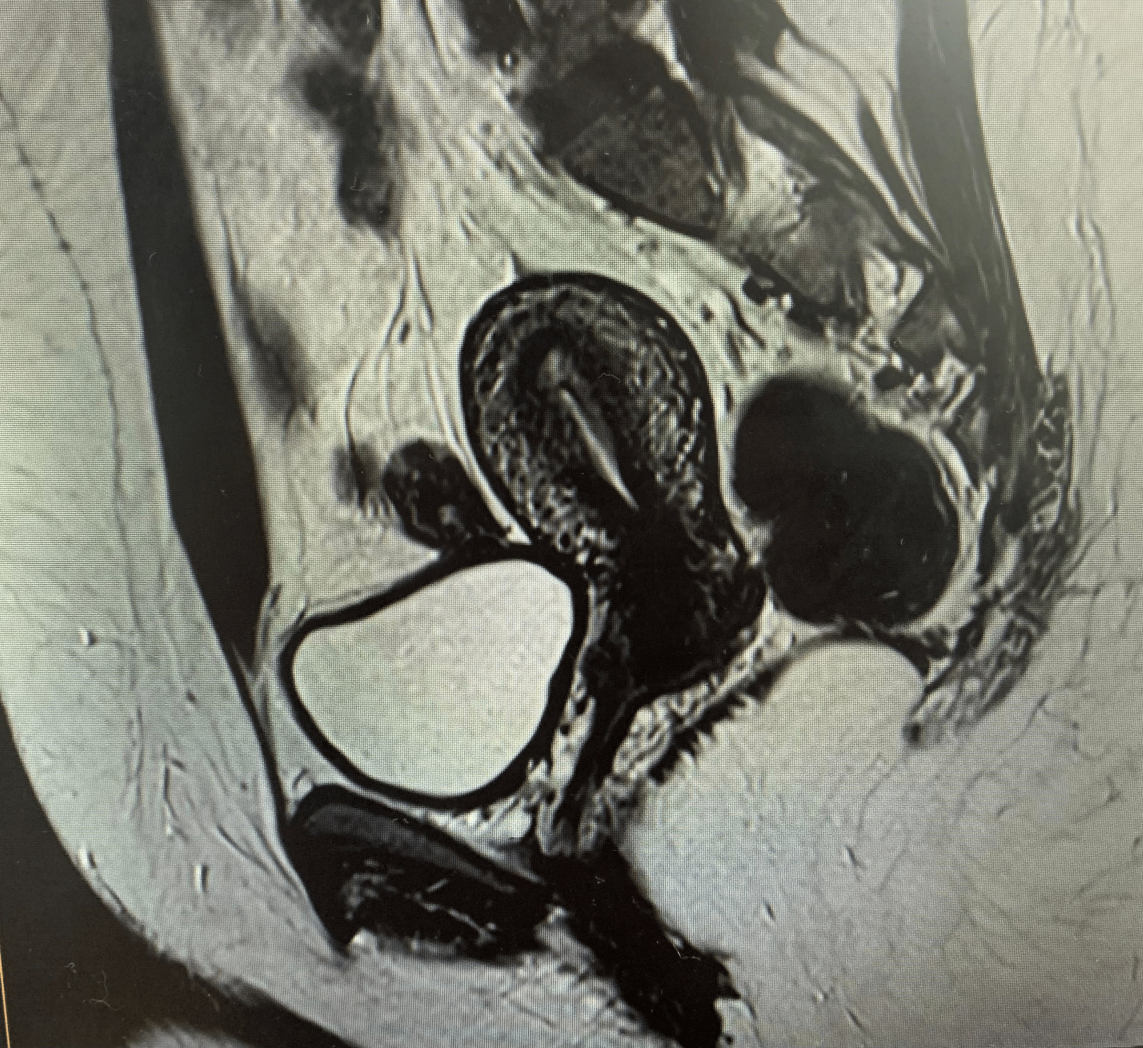
Postoperative MRI sagittal pelvic imaging MRI: Magnetic resonance imaging

Surgical excision was performed via an anterior vaginal wall incision. Figure 2 shows postoperative MRI sagittal pelvic imaging. Grossly, the lesion appeared as a calcified fibroid. Histopathology showed fragments of squamous-lined mucosa with underlying proliferation of spindle and epithelioid cells in a collagenous stroma, admixed with foamy histiocytes and lymphocytes. No atypia, necrosis, or significant mitotic activity (<1/10 HPFs) was observed. Immunohistochemistry demonstrated diffuse positivity for ALK, CD68, CD163, and factor XIIIa; patchy positivity for CD10; focal positivity for smooth muscle actin (SMA); and negativity for CD34, desmin, h-caldesmon, S100, SOX10, melan A, HMB45, ER, PR, AE1/3, epithelial membrane antigen (EMA), myogenin, and MyoD1. Overall, the features were consistent with an ALK-positive mesenchymal tumor.

Following histopathology, the patient was referred to the gynecologic oncology team. CT of the abdomen and pelvis showed mildly enlarged right ileocolic lymph nodes, likely due to mesenteric adenitis, with no other abnormalities. MRI confirmed no evidence of recurrence or residual disease. During the multidisciplinary discussion, considering the complete excision, negative imaging, and the risks associated with re-excision near the bladder trigone, surveillance was recommended. The patient was advised on self-examination, scheduled for clinical follow-up every three months, and planned for periodic imaging to monitor for recurrence, in line with sarcoma surveillance protocols.

## Discussion

Primary vaginal tumors are exceptionally rare, accounting for merely 1-2% of all female genital tract malignancies, with mesenchymal tumors constituting an even smaller proportion [3]. The majority of vaginal masses encountered in clinical practice are benign, such as leiomyomas, Gartner duct cysts, or endometriotic nodules, which frequently occur on the anterior wall of the vagina. In light of this, the occurrence of an ALK-positive mesenchymal tumor in the vaginal region is exceedingly uncommon and warrants a comprehensive description.

In this case, a 20-year-old woman presented with a progressively enlarging mass on the anterior aspect of her vaginal wall, initially misdiagnosed as a fibroid. This diagnostic challenge corroborates previous research indicating that radiological evaluations prior to surgery frequently suggest benign conditions in patients with vaginal mesenchymal tumors [7]. Although MRI assists in delineating the extent of the lesion, it lacks sufficient accuracy to differentiate among IMTs, ALK-positive tumors, and other spindle-cell lesions. This underscores the critical role of histopathology and immunohistochemistry in establishing a definitive diagnosis [8].

Histologically, the proliferation of spindle and epithelioid cells in a collagenous stroma with associated lymphocytes and histiocytes, combined with ALK immunoreactivity, established the diagnosis of an ALK-positive mesenchymal tumor [9].

Reports of ALK-positive mesenchymal tumors within the female genitourinary tract are exceedingly rare. To date, only isolated instances have been documented, predominantly observed in infants, adolescents, and young women, thereby underscoring a potential inclination toward occurrence in younger populations [10-13]. Fuehrer et al. (2012) documented eight cases of ALK-positive IMTs in various genital tract sites, demonstrating ALK immunopositivity in seven and gene rearrangements in five, confirming the pathogenetic role of ALK activation [9]. Similarly, Pickett et al. (2017) identified ALK-positive IMTs in 6 of 1,747 uterine tumors previously misclassified as smooth muscle neoplasms, representing 0.3% of “leiomyomas” and 2.3% of “leiomyosarcomas,” emphasizing the potential for diagnostic overlap [14]. Devereaux et al. (2019) further reported ALK rearrangements in 14% of uterine and cervical tumors initially labeled as smooth muscle tumors of uncertain malignant potential [15]. Collectively, these findings highlight the diagnostic value of ALK immunostaining and molecular analysis in differentiating IMTs from morphologically similar mesenchymal tumors of the female genital tract, supporting the inclusion of ALK testing in ambiguous cases such as the present one.

Therapeutically, complete surgical removal is regarded as the gold standard, with the extent of resection tailored to the anatomical site and associated risks. In this instance, a complete local excision was feasible. Consequently, a multidisciplinary consensus endorsed surveillance, supported by negative postoperative imaging.

## Conclusions

This case contributes to the limited research on ALK-positive mesenchymal tumors of the female genital tract, and, to our knowledge, is one of the few reported in the vagina. It raises three key clinical points: first, young women with anterior vaginal wall masses should consider mesenchymal tumors in their diagnosis, despite how rare they are; second, histopathology and immunohistochemistry are vital for distinguishing these tumors from their benign mimics; and third, personalized management plans, developed through multidisciplinary discussion, are essential for striking a balance between cancer control and preserving function.
